# A transcriptome approach towards understanding the development of ripening capacity in ‘Bartlett’ pears (*Pyrus communis* L.)

**DOI:** 10.1186/s12864-015-1939-9

**Published:** 2015-10-09

**Authors:** Ngoc T. Nham, Sergio Tonetto de Freitas, Andrew J. Macnish, Kevin M. Carr, Trisha Kietikul, Angelo J. Guilatco, Cai-Zhong Jiang, Florence Zakharov, Elizabeth J Mitcham

**Affiliations:** Department of Plant Sciences, University of California, Davis, CA 95616 USA; Present address: Embrapa Tropical Semi-Arid, Petrolina, PE 56302-970 Brazil; Present address: Horticulture and Forestry Science, Queensland Department of Agriculture, Fisheries and Forestry, Maroochy Research Facility, Nambour, QLD 4560 Australia; Research Technology Support Facility, Michigan State University, East Lansing, MI 48824 USA; Agriculture Research Service, United States Department of Agriculture, Davis, CA 95616 USA

**Keywords:** RNA-Seq, EBSeq, Cell wall, Auxin, Ethylene, bZIP, AP2/EREBP, bHLH, WRKY, Aux/IAA

## Abstract

**Background:**

The capacity of European pear fruit (*Pyrus communis* L.) to ripen after harvest develops during the final stages of growth on the tree. The objective of this study was to characterize changes in ‘Bartlett’ pear fruit physico-chemical properties and transcription profiles during fruit maturation leading to attainment of ripening capacity.

**Results:**

The softening response of pear fruit held for 14 days at 20 °C after harvest depended on their maturity. We identified four maturity stages: S1-failed to soften and S2- displayed partial softening (with or without ET-ethylene treatment); S3 - able to soften following ET; and S4 - able to soften without ET. Illumina sequencing and Trinity assembly generated 68,010 unigenes (mean length of 911 bp), of which 32.8 % were annotated to the RefSeq plant database. Higher numbers of differentially expressed transcripts were recorded in the S3-S4 and S1-S2 transitions (2805 and 2505 unigenes, respectively) than in the S2-S3 transition (2037 unigenes). High expression of genes putatively encoding pectin degradation enzymes in the S1-S2 transition suggests pectic oligomers may be involved as early signals triggering the transition to responsiveness to ethylene in pear fruit. Moreover, the co-expression of these genes with *Exps* (*Expansins*) suggests their collaboration in modifying cell wall polysaccharide networks that are required for fruit growth. K-means cluster analysis revealed that auxin signaling associated transcripts were enriched in cluster K6 that showed the highest gene expression at S3. *AP2/EREBP* (*APETALA 2/ethylene response element binding protein*) and *bHLH* (*basic helix-loop-helix*) transcripts were enriched in all three transition S1-S2, S2-S3, and S3-S4. Several members of Aux/IAA (Auxin/indole-3-acetic acid), ARF (Auxin response factors), and WRKY appeared to play an important role in orchestrating the S2-S3 transition.

**Conclusions:**

We identified maturity stages associated with the development of ripening capacity in ‘Bartlett’ pear, and described the transcription profile of fruit at these stages. Our findings suggest that auxin is essential in regulating the transition of pear fruit from being ethylene-unresponsive (S2) to ethylene-responsive (S3), resulting in fruit softening. The transcriptome will be helpful for future studies about specific developmental pathways regulating the transition to ripening.

**Electronic supplementary material:**

The online version of this article (doi:10.1186/s12864-015-1939-9) contains supplementary material, which is available to authorized users.

## Background

European pears (*Pyrus communis* L.), including ‘Bartlett’, ‘d’Anjou’, and ‘Comice’, are economically significant fruit in the United States, with a production value of $437 million in 2012 [1]. As a climacteric fruit, pears ripen in association with a substantial increase in rates of respiration and ethylene biosynthesis [2]. Unlike many climacteric fruit such as apple and mango, European pears develop poor texture and flavor if left to ripen on the tree [3]. Therefore, most European pears are harvested at the mature-green stage and then usually exposed to ethylene or cold temperatures (e.g., −1 to 10 °C) prior to ripening to enhance their ability to produce ethylene and ripen at 20 °C [4]. Hansen found that early maturity ‘Bartlett’ and ‘d’Anjou’ pear might not respond to ethylene or cold treatment while late maturity fruit could ripen without any conditioning treatment [5]. However, the underlying molecular mechanisms governing this developmental shift are still not well understood. Furthermore, as a climacteric fruit, pear fruit ripening includes the transition from auto-inhibitory ethylene (also known as “System 1”) to autocatalytic ethylene (“System 2”) that regulates the numerous metabolic processes associated with fruit ripening [6]. The intrinsic developmental factors that regulate the transition from System 1 to System 2 remain mostly unknown [6].

Ripening is postulated to be initiated by activation of specific transcriptional regulators, such as *colorless non ripening* (*CNR*) and *ripening-inhibitor* (*RIN*), as first identified in tomato, a model organism to study fruit ripening. These regulators lead to signal transduction pathways that include ethylene as an essential signaling molecule [7, 8]. These signaling pathways control many ripening-related biochemical events such as chlorophyll degradation, starch degradation to sugars, decreases in organic acids, and production of aroma compounds [6, 7, 9]. Several studies designed to elucidate the molecular pathways of fruit ripening have focused on genes associated with hormone and cell wall metabolism, as well as transcriptional regulation [8, 10, 11].

Some of the molecular aspects of European pear ripening have been investigated [4]. Several studies reported an increase in ethylene biosynthesis enzymes, 1-aminocyclopropane-1-carboxylate (ACC) synthase and ACC oxidase, following ethylene treatment and cold storage [12–14]. Increases in transcript abundance of pear fruit ethylene biosynthesis genes (e.g., *Pc-ACS1b* and *Pc-ACS2b*) [15] and ethylene perception genes including *Pc-ETR1a* and *Pc-ERS1a* [16] during fruit ripening were also reported. Low transcript abundance of genes encoding cell wall modifying proteins such as *β-galactosidases* and *expansins* were detected during fruit development in ‘Rocha’ pear [17]. In addition, large-scale expression profiles of ‘Rocha’ and ‘La France’ pear during fruit growth and ripening have been generated [17, 18]. However, these two studies utilized microarrays with a limited number of fruit-specific sequences. To our knowledge, genes associated with hormones other than ethylene and transcription factors have not been characterized during pear fruit development.

In the last 5 years, next generation sequencing (NGS) technologies accompanied by sophisticated bioinformatics tools have been developed and provide a powerful approach to examine the transcriptomes of non-model plants [19, 20]. Accordingly, these tools have been utilized to determine transcriptional changes during fruit growth and development in a variety of species including Chinese bayberry (*Myrica rubra*) [21], orange (*Citrus sinensis*) [22], and Korean black raspberry (*Rubus coreanus*) [23].

In the present study, NGS technology was used to characterize the molecular mechanisms regulating the development of ripening capacity in ‘Bartlett’ pear fruit. The specific objectives were to 1) develop a better understanding of the acquisition of pear ripening capacity and 2) define the molecular regulation of pear fruit ripening, focusing on genes associated with cell wall metabolism, hormone biosynthesis and signaling, and transcription factors.

## Methods

### Plant materials and physico-chemical analysis

‘Bartlett’ pear fruit were produced at a commercial orchard in Sacramento County, California, USA. Fruit were harvested at 7-day intervals for 4 weeks, from 100 to 120 DAFB; the fourth harvest time was equivalent to the first commercial harvest. Sixty fruit were collected at each harvest time from a total of five trees. Immediately after harvest, fruit were randomized and divided into five groups of 12. Each group was composed of three biological replications with four fruit each. Group 1 fruit were analyzed within 24 h of harvest for ethylene production rate, respiration rate, weight, diameter, skin color, flesh firmness, and SSC. Group 2 fruit were used to measure the internal ethylene concentration. Peel tissues for molecular analysis were collected from fruit in Group 3. Fruit from Groups 4 and 5 were enclosed in separate 20 L glass jars and treated with 0 or 100 μLL^−1^ ethylene in flowing air streams of 1500 mLmin^−1^ for 24 h at 20 °C. These fruit were then held at 20 °C and 90 % relative humidity for 14 days to allow for ripening. After 14 days (D14), fruit were evaluated for skin color, flesh firmness, and SSC.

Rates of ethylene production and respiration were assessed for each replication by sealing four fruit inside a 3.8 L glass jar and using the method described by Villalobos et al. [24]. Headspace samples were collected with 10 mL syringes and injected into a gas chromatograph for ethylene quantification (Model Carle AGC-211, EG&G Chandler Engineering, Tulsa, OK) or a PIR-2000R infrared analyzer for CO_2_ analysis (Horiba Instruments Inc., Irvine, CA).

Fruit diameter was measured across the widest point of each fruit with a caliper. Pear skin color was determined on two diametrically opposite sides of each fruit using a Chroma Meter CR-310 (Minolta Ltd., Osaka, Japan). The color data were captured using the CIE 1976 (L*, a*, b*) color space and expressed as the hue angle (h°), where 90° represents full yellow and 180° corresponds to full green. Flesh firmness was quantified as the resistance to 9 mm penetration with an 8 mm-diameter probe using a Fruit Texture Analyzer (Güss, Strand, South Africa) on two opposite sides of the fruit after the peel was removed. SSC was measured in juice samples extracted by squeezing cortical wedges cut from two opposite sides of each of four fruit in two layers of cheesecloth, with a Reichert AR6 Series refractometer (Reichert Inc., Depew, NY).

The internal ethylene concentration was determined according to Coombe and Hale [25] and Chervin et al. [26]. Briefly, pre-weighed fruit were placed individually in a chamber containing a saturated solution of NaCl. Each fruit was submerged in the solution under an inverted funnel with the narrow end capped with a rubber septum. The air trapped in the narrow end of the funnel was withdrawn with a syringe. The chamber was sealed and a partial vacuum of −700 mm Hg was applied for 5 min. After returning to atmospheric pressure, 1 mL of the fruit internal atmosphere trapped in the narrow end of the funnel was sampled by syringe and the ethylene concentration was determined by gas chromatography as described above.

Statistical analysis was performed on each variable by means of analysis of variance using the SAS statistical package (Version 9.1, SAS Institute Inc., Cary, NC). The mean values of three replications were compared using Tukey’s test (p-value ≤ 0.05).

### RNA extraction

Total RNA was isolated from 0.5 g tissues ground in liquid N_2_, which contained both skin and flesh tissues peeled from two opposite sides of 4 fruit (Group 3 from the four harvest times), using the Qiagen RNeasy Plant Mini Kit (Qiagen, Limburg, Netherlands) according to the manufacturer’s instructions. The total RNA was then treated with DNase I recombinant, RNase-free (Roche, Basel, Switzerland) to remove DNA contamination. The total RNA concentration was quantified using a NanoDrop spectrophotometer (Thermo Fisher Scientific, MA), with absorbance at 260 nm. The quality of total RNA was verified by examining the ratio OD260/OD280 and formaldehyde agarose gel electrophoresis.

### RNA sequencing

Illumina library preparation and sequencing of 12 samples (four harvest times X three biological replicates) were completed following standard protocols at the UC Davis DNA Technologies Core (http://dnatech.genomecenter.ucdavis.edu/). The integrity and quantity of total RNA was examined using an Agilent 2100 Bioanalyzer RNA 6000 kit and Invitrogen’s Qubit. mRNA was isolated from total RNA using Dynabeads oligo-d(T)_25_ (Invitrogen, Life Technologies, CA). The RNA-Seq library was constructed by following the TruSeq protocol (Illumina Inc., San Diego, CA). Individual libraries were prepared with barcodes and pooled for sequencing on one lane of the Illumina HiSeq 2000 platform. Paired-end reads of 100 cycles were collected and fastq files were generated using the Illumina pipeline.

### *De novo* assembly and count estimation

Given that inclusion of a greater number of reads in *de novo* assembly produces a greater contiguity of sequences [27], Illumina reads obtained from this experiment (12 RNA samples) and a second ‘Bartlett’ pear ripening capacity experiment (9 RNA samples) were combined for the assembly. The raw reads were trimmed to remove TruSeq adapters and low quality bases, using Trimmomatic (v0.22) [28]. Surviving paired reads were used as input for *de novo* transcript assembly. The assembly was carried out using Trinity (ver. trinityrnaseq_r2012-06-08) [29] with default parameters except --min_kmer_cov was set to 3. To minimize redundancy in the set of putative transcripts, the contigs were clustered using CD-HIT [30, 31] and then with TGICL [32]. Stringent similarity parameters were selected to minimize the likelihood of merging paralogous transcripts. This reduced the number of contigs in the original output by Trinity. As these contigs may still represent multiple isoforms of the same gene, contigs that shared a common Trinity component and sub-component were naively grouped into unigenes by RSEM (v1.1.21) [33]. Estimated read counts associated with the assembled contigs were determined with RSEM, which utilizes Bowtie to map reads to a reference database composed of the assembled contigs [34]. In preparing this database the unigene to contig mapping described above was provided to permit RSEM to estimate read counts at both the individual contig (putative isoforms) and unigene level. The RSEM output represented the estimated counts of reads associated with each isoform or unigene, recognizing the uncertainty inherent in assigning reads to isoforms that may share one or more exons.

### Sequence identity validation and quantitative PCR validation

Sequences in the *de novo* transcriptome were mapped to the reference genome of Asian pear (*P. bretschneideri*) [35] and European pear (*P. communis*) [36] using GMAP (v. gmap-gsnap-2013-07-20) [37] to check for possible chimeric and non-match sequences, using k-mer 13. ORFs were examined using OrfPredictor [38] with an ORF cut-off length of 200 base pairs. The BLASTX program (v2.2.26+) [39] was used to perform similarity searches of the contigs against the TAIR v10 and RefSeq (v54, plant only) protein databases with an e-value threshold of 1e^−10^. The contigs were annotated with the description inherited from the best hits in each database.

cDNA was synthesized from 1 μg DNase - treated total RNA, using Superscript™ III First Strand Synthesis Systems for RT-PCR Systems (Invitrogen, Life Technologies, CA). Before qPCR validation, sequences of interest were aligned against the available sequences of Asian and European pears on the NCBI EST database and their published genome to confirm sequence identity, using the local tblastn function in BioEdit (v7.1.3.0) [40]. The gene expression was examined using SYBR Green PCR Master Mix and a 7300 Real Time PCR System (Applied Biosystems, Life Technologies, CA). *Ef1alpha* was chosen as the housekeeping gene after testing with *18 s, 26 s, β-actin,* and *tubulin1*. Primers for sequences of interest were designed using Primer 3 [41, 42] and passed the primer efficiency check for qPCR. In the regression analysis, the FC of qPCR was ΔΔCt [43] and the FC of RNA-Seq was the base-2 logarithm of the ratio RSEM count in treatment 2/RSEM count in treatment 1.

### Mapman functional annotation analysis

Mapman functional annotation analysis was utilized to gain an understanding about the general function of genes expressed during fruit growth and to identify gene families that may play essential roles in regulating the development of pear ripening capacity. Contigs were classified into specific functional groups, using Mercator [44] with a blast cut-off of 50. Because one unigene might have multiple contigs, a functional term of a unigene was derived from its representative contig that had the highest bit score. Enrichment analysis was completed through Fisher’s test using Mefisto (http://www.usadellab.org/cms/index.php?page=mefisto) with Bonferroni correction. Gene expression changes were viewed in Mapman 3.5.1R2 [45].

### Differential expression analysis

The unigene counts were subjected to both pairwise and multi-condition analysis to detect DE sequences between two harvest times and among four harvest times of pear fruit, respectively, using the EBSeq package (v1.1.6) with False Discovery Rate of 0.05 [46]. The method employed by EBSeq manages the varying uncertainty in counts across isoform groups. In convergence checking, the maximum round of each comparison was chosen based on a difference less than 0.001 between the two last iterations of EBOut$Alpha, and of EBOut$Beta (N. Leng, personal communication, 2013). For pairwise analysis, unigenes with a posterior probability of being differentially expressed (PPDE) of ≥ 0.95 were identified as differentially expressed between two harvest times. For multi-condition analysis, unigenes with P1 ≤ 0.05 (P1 is the probability that unigenes are equally expressed among four stages of development) were identified as differentially expressed across the four harvest times. Normalized counts of unigenes for calculating gene fold changes were obtained from the multi-condition analysis.

### K-means cluster analysis

K-means clustering was utilized to determine particular patterns in gene expression throughout the four harvest times, using the base-2 logarithm of the average normalized counts of three biological replicates. The number of clusters was identified using the Figures of Merit application embedded in MEV [47]. Unigenes in each cluster were then identified using the R package amap (http://cran.r-project.org/web/packages/amap/index.html) with Pearson correlation, in which 100 random sets were applied to generate reproducible clusters. A heatmap of the number of unigenes in Mapman categories in each cluster was built on the R package gplots (http://cran.r-project.org/web/packages/gplots/index.html).

### Accession code

The clean reads produced in this study have been deposited at DDBJ/EMBL/GenBank Short Read Archive: 12 BioSample numbers SAMN02929682 - SAMN02929693, 12 accession codes SRR1572168 - SRR1572991, and under project number PRJNA255920. This Transcriptome Shotgun Assembly project has been deposited at DDBJ/EMBL/GenBank under the accession GBXL00000000. The version described in this paper is the first version, GBXL01000000. The gene ID, contig ID, and their putative function can be accessed through Additional file 8.

### Computer system

Except for the *de novo* transcriptome assembly and mapping to a reference genome, all data analyses were completed with a Dell Optiplex 390 4GB RAM, 32-bit, Intel(R) Core(TM) i5-2400 CPU with Windows 7 Enterprise, Microsoft Office 2000, and R 2.15.0 (The R Core Development Team, 2013), RStudio i386-pc-mingw32/i386 platform.

## Results and discussion

### Physico-chemical changes during fruit growth and development

**Fig. 1:**
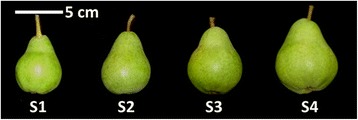
Pear fruit at four harvest times.S1: 100 DAFB, S2: 106 DAFB, S3: 113 DAFB, and S4: 120 DAFB (S4 corresponded to the first commercial harvest date of the season)

To characterize the relationship between pear fruit maturation and the development of ripening capacity, ‘Bartlett’ pear fruit were harvested at weekly intervals commencing 3 weeks before commercial harvest to capture four progressive stages of maturity: S1: 100 days after full bloom (DAFB), S2: 106 DAFB, S3: 113 DAFB, and S4: 120 DAFB (S4 corresponded to the first commercial harvest date of the season) (Fig. 1). Fruit growth and maturity were monitored by physico-chemical measurements (Table 1). Fruit weight and diameter at harvest increased considerably with these advancing stages of maturity (Table 1). In contrast, the flesh firmness at harvest steadily decreased as fruit maturity increased (Table 1). Rates of respiration and the internal ethylene concentration were highest in fruit harvested at S1 and relatively low in S2, S3, and S4 (Table 1). Rates of ethylene production were also relatively higher in S1 than S2 and S3 before increasing again at S4 (Table 1). Despite the higher ethylene production rate at S1 (0.128 μLkg^−1^h^−1^), the level was substantially lower than typical rates produced during climacteric ripening of ‘Bartlett’ pear, which can be as high as 150 μLkg^−1^h^−1^ [13, 14, 24]. There were no significant differences in fruit soluble solids content (SSC) and skin color among four harvest maturity stages examined in this study (Table 1).

**Table 1:** Physico-chemical analysis of ‘Bartlett’ pear fruit at four harvest times

Maturity stage	Weight (g)	Diameter (mm)	Firmness (N)	SSC (%)	Skin Color (h°)	Respiration CO_2_ (mgkg^-1^h^-1^)	Ethylene	
							Internal (nLL^-1^g^-1^)	Production (μLkg^-1^h^-1^)
S1	83.9 d^a^	52.1 c	121.6 a	9.7 a	116.8 a	40.6 a	0.41 a	0.128 a
S2	102.9 c	55.1 c	111.1 ab	10.9 a	116.7 a	25.3 bc	0.15 b	0.037 c
S3	128.2 b	60.6 b	100.9 b	10.2 a	117.2 a	31.1 b	0.12 b	0.078 b
S4	187.7 a	67.6 a	86.5 c	11.1 a	116.5 a	20.5 c	0.19 b	0.113 a

^a^Mean values with different letters are significantly different according to Tukey’s test (p-value ≤ 0.05)

**Fig. 2:**
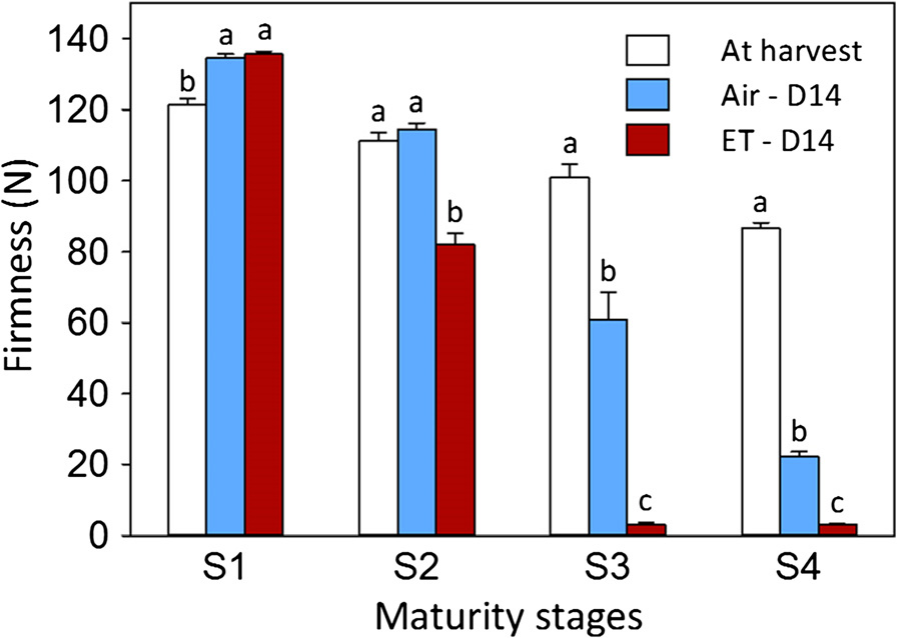
Firmness changes of pears harvested at four maturity stages at harvest and after air/ethylene treatment. S1, S2, S3, and S4 were harvested a week apart; S4 coincident with commercial harvest (RNA extracted from peel tissues of S1 to S4 at harvest were used for RNA sequencing). D14: 14 days at 20 °C following treatment of pears with air or 100 μLL^−1^ ethylene (ET) for 24 h. Bars indicate standard errors; letters indicate significant differences among the three firmness values within a stage according to Tukey’s test (p-value ≤0.05)

After harvest, fruit at each of the four maturity stages were treated with 0 or 100 μLL^−1^ ethylene for 24 h and then evaluated for their ripening capacity based on softening after being held at 20 °C for 14 days. The ability of fruit to soften in the presence or absence of ethylene increased with advancing harvest maturity (Fig. 2). When treated with ethylene, S1 fruit failed to soften, S2 fruit displayed partial softening (from 111.1 N to 81.8 N), and S3 and S4 fruit softened to a firmness of <5 N. In the absence of ethylene treatment, fruit harvested at stages S1 and S2 failed to soften, while S3 and S4 fruit softened to 60 N and 22.1 N, respectively. Therefore, it appears that the slightly higher rate of ethylene production during the preclimacteric phase in S1 had no positive effect on the ability of fruit to ripen when harvested at this stage. The slight increase in firmness observed for S1 fruit at day 14 shelf life presumably reflected water loss during storage of fruit harvested at an immature stage; this agrees with what has been found in apple and bell pepper [48, 49]. The general fruit softening response was also accompanied by similar changes in peel color, as evidenced by the hue angle (h°) (Additional files 1 and 2). There was no significant effect of harvest maturity and ethylene treatment on fruit SSC at the completion of a 14-day shelf life (Additional file 1).

In other species, standard stages of fruit development have been well established. For instance, these stages in tomato include Green, Mature Green, Breaker, Pink, and Red Ripe [7, 8], while peach and plum development is described as an S1 to S4 double sigmoid pattern [50, 51]. For ‘Bartlett’ pear, we are unaware of defined standard stages of fruit development, except those utilizing firmness as a ripeness indicator: 85–98 N, when the fruit are ready to harvest [52] and 20 N, when the fruit are ready to consume [53], with a consideration of SSC (≥10 %) and size (≥60.3 mm) [52]. In the current study, because of the low ripening capacity of pear fruit at early maturity stages, we considered full ripening capacity was achieved when fruit firmness reached 20 N after 14 days at 20 °C; this was named “RC14” for “Ripening Capacity at 14 days” after harvest. Given this definition of ripening capacity, we observed the following response of the four harvest maturity stages: S1 and S2 did not achieve RC14; S3 achieved RC14 with ethylene treatment; and S4 fruit achieved RC14 without ethylene treatment.

### RNA-Seq and *de novo* assembly

RNA sequencing of the peel tissue of the four maturity stages (S1 to S4 at harvest) generated 187.3 million (mil) 2x100 bp paired-end reads. Of the 357.6 mil paired-end reads from both experiments, 81.7 % were retained after the quality check, in which the unqualified read was mostly due to Bottom Middle Swath in the sequencing system. A Trinity assembly on 292 mil qualified paired-end reads generated 101,109 contigs that were clustered into 68,010 unigenes. The contig length ranged from 201 to 18,868 bp, with a median length of 502 bp and a mean length of 911 bp.

### Validation of the transcriptome in sequence identity and expression levels

**Fig. 3:**
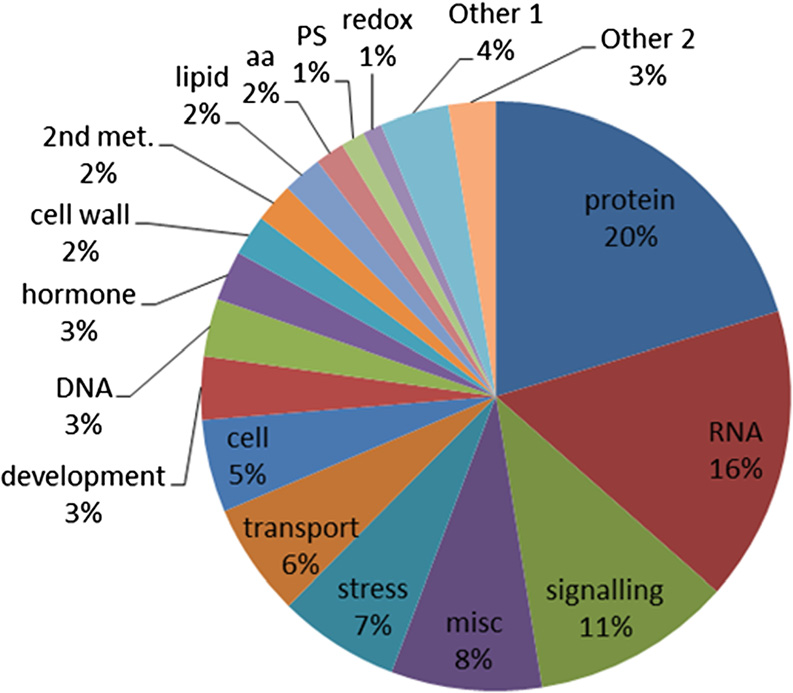
Mapman functional annotations of the transcriptome. 2nd: secondary, aa: amino acid, PS: photosynthesis, met.: metabolism, syn: synthesis, Other 1: nucleotide met., minor CHO met., major CHO met., mitochondrial electron transport/ATP and glycolysis; Other 2: co-factor and vitamin met., TCA/org transformation, metal handling, tetrapyrrole syn., C1-met., OPP, N-met., biodegradation of xenobiotics, fermentation, polyamine met., gluconeogenesis/glyoxylate cycle, S-assimilation, micro RNA and natural antisense

Sequence identity of the *de novo* transcriptome was first validated through putative function determination. BLASTX of the contigs against the NCBI RefSeq (v54 plant only) and Arabidopsis (TAIR10) protein databases identified similar proteins (with a threshold e-value of 1e^−5^) in these reference sets for 40.6 % and 31.7 %, respectively, of the 68,010 unigenes. This indicates that the functions of a large portion of the genes of *P. communis* have not yet been identified. Using the NCBI non-redundant database with a threshold of 1e^−5^, 68 %, 80 %, and 93 % of unigenes of Chinese bayberry, Korean black raspberry, and ‘Suli’ pear transcriptomes, respectively, were annotated [21, 23, 54]. In the general functional description of the transcriptome examined using Mapman, 22.3 % of the unigenes were assigned to 34 meaningful bincodes of Mapman, with the highest numbers of unigenes classified into Protein (20 %), RNA (16 %), Signaling (11 %), Stress (7 %), and Transport (6 %) categories (Fig. 3).

Open reading frame (ORF) finders evaluate the degree to which full coding sequence are assembled [55]. This analysis determined 55,917 (55.3 %) contigs had an ORF of length ≥ 200 bp, with an average length of 724 bp. Moreover, the high percentages of mapped contigs when mapping to reference genomes indicated good sequence identity of our *de novo* transcriptome. Mapping all contigs of the *de novo* transcriptome to the Asian pear (*P. bretschneideri*) genome [35] revealed that 95,960 (94.9 %) were mapped to the reference genome, in which 5,554 (5.5 %) contigs were possibly chimera sequences, and 5,149 (5.1 %) contigs were non-matched sequences. Additionally, mapping to the recently published European pear (*P. communis*) genome [36] showed 99,602 (98.5 %) mapped contigs, in which 9,096 (9.0 %) were possible chimeras, and 1,507 (1.5 %) were non-matched sequences.

**Fig. 4:**
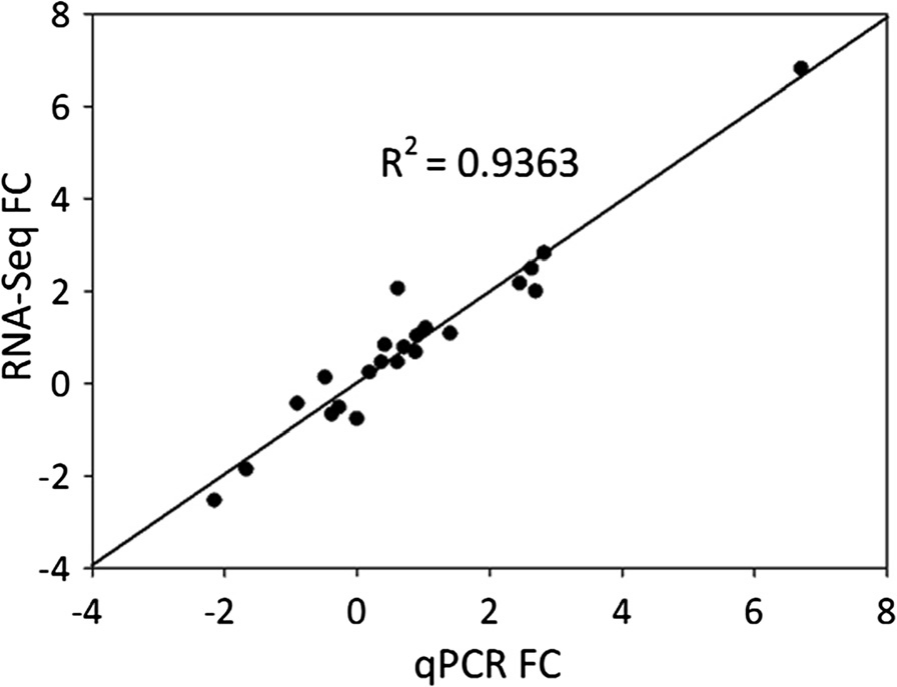
Regression analysis of gene expression fold changes (FC) obtained from quatitative PCR and RNA-Seq (p-value <0.001)

To validate gene expression values obtained from RNA-Seq data, we examined the correlation between fold changes (FCs) calculated on RSEM (RNA-Seq by Expectation Maximization) counts [33] and the equivalent values measured by quantitative PCR (qPCR). The validation on eleven transcripts associated with cell wall metabolism, hormone biosynthesis and signaling, and transcriptional regulation (Additional file 3) yielded an R^2^ of 0.9363 (p-value < 0.001) (Fig. 4). The correlation was stronger than those recently published for ‘Suli’ pear (R^2^ = 0.75) [54] and for Chinese bayberry (R^2^ = 0.83) [22]. This analysis confirmed the reliability of the gene expression values generated from RNA-Seq.

### Multi-condition and pairwise differential expression analysis

**Fig. 5:**
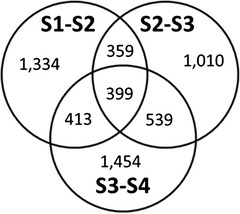
Unique and shared differentially expressed unigenes in S1-S2, S2-S3, and S3-S4 pairwise analysis

Differential expression analysis was conducted comparing multiple treatments or two treatments (pairwise analysis) [46]. The analysis on all four harvest maturity stages generated 7,015 unigenes that were significantly different across these stages. The results of the pairwise analysis on two maturity stages are presented in Table 2. The increased number of significant differentially expressed (DE) unigenes from 2,505 between S1 and S2, to 3,397 between S1 and S3, and to 4,785 between S1 and S4 suggests there were fewer transcriptional differences between closer stages. Regarding the transition between two adjacent stages, fewer gene expression changes occurred during the S2-S3 transition, when fruit gained the ability to soften to 20 N after ethylene treatment, than during the earlier S1-S2 transition, when fruit failed to ripen, and the later S3-S4 transition, when fruit developed the capacity to soften without ethylene treatment. Moreover, the highest number of DE unigenes in the S3-S4 transition (2,805) suggests sophisticated molecular mechanisms occurred during this transition. Further analysis identified which DE unigenes between two adjacent maturity stages were unique or shared across the three transitions (Fig. 5). A total of 399 DE unigenes were shared across all three transitions. The function of selected shared DE genes, along with unique DE unigenes, as related to fruit maturity and ripening capacity development is discussed later in this paper.

**Table 2:** Number of differentially expressed unigenes between two maturity stages

Maturity stage	S1	S2	S3	S4
S1	NA			
S2	2505	NA		
S3	3397	2037	NA	
S4	4785	3105	2805	NA

NA: not applicable

### K-means clusters and functional annotation analysis of the clusters

K-means clustering revealed representative patterns of gene expression over the four harvest maturity stages. Because we considered these patterns across four maturity stages, K-means clustering was processed on the 7,015 DE unigenes generated from multi-condition differential expression analysis, instead of the DE unigenes from pairwise comparison. Using Mapman classification, 68.5 % of 7,015 DE unigenes were assigned to the 34 functional groups, while only 22.1 % of total unigenes were assigned to these groups (Additional file 4). This indicates that a large portion of DE unigenes associated with the four maturity stages had their putative functions identified and they could be visualized using Mapman.

**Fig. 6:**
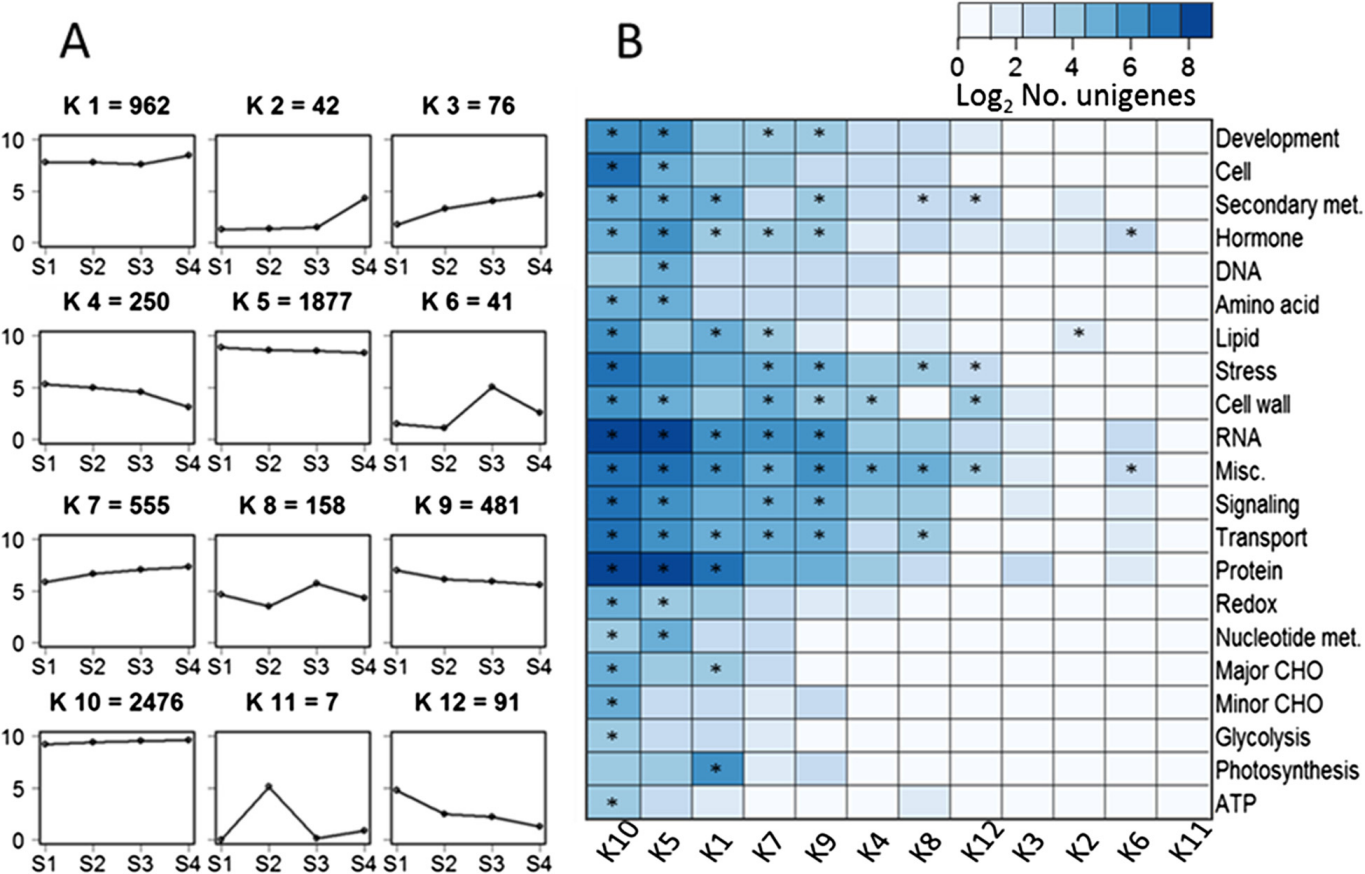
K-means clustering for differentially expressed unigenes across four stages (**a**) and functional classification within clusters (**b**). **a** The y-axis is the base-2 logarithm of the mean of normalized counts of three biological replicates. The number of unigenes in each cluster is indicated. **b** Clusters from left to right: from the most abundant K10 to the least abundant K11. * indicates enriched categories according to Fisher’s test (p-value ≤0.05)

Twelve clusters containing between 7 and 2476 unigenes were determined (Fig. 6a). Of the considered unigenes, 44.9 % fell into four clusters (K2, K3, K7, and K10) that increased in transcript abundance from S1 to S4 while 38.5 % belonged to four clusters (K4, K5, K9, and K12) that decreased in expression. Clusters K11 and K2 contained unigenes that were strongly expressed at the S2 and S4 stages, respectively. However, no enriched categories were identified in K11 and the enriched categories in K2 were not associated with our functional groups of interest, including cell wall metabolism, hormone biosynthesis and signaling, and transcription factors (Fig. 6b). Clusters K6 and K8 showed high expression of unigenes at S3. The Aux/IAA transcription factor family was enriched in both of these clusters, and the auxin-associated functional group was enriched in K6 (Fisher’s test, p-value ≤ 0.05) (Additional file 5). This suggests that the auxin-associated transcripts may play an important role in the S2-S3 transition.

### Expression change in genes associated with cell walls, hormones, and transcription factors

**Fig. 7:**
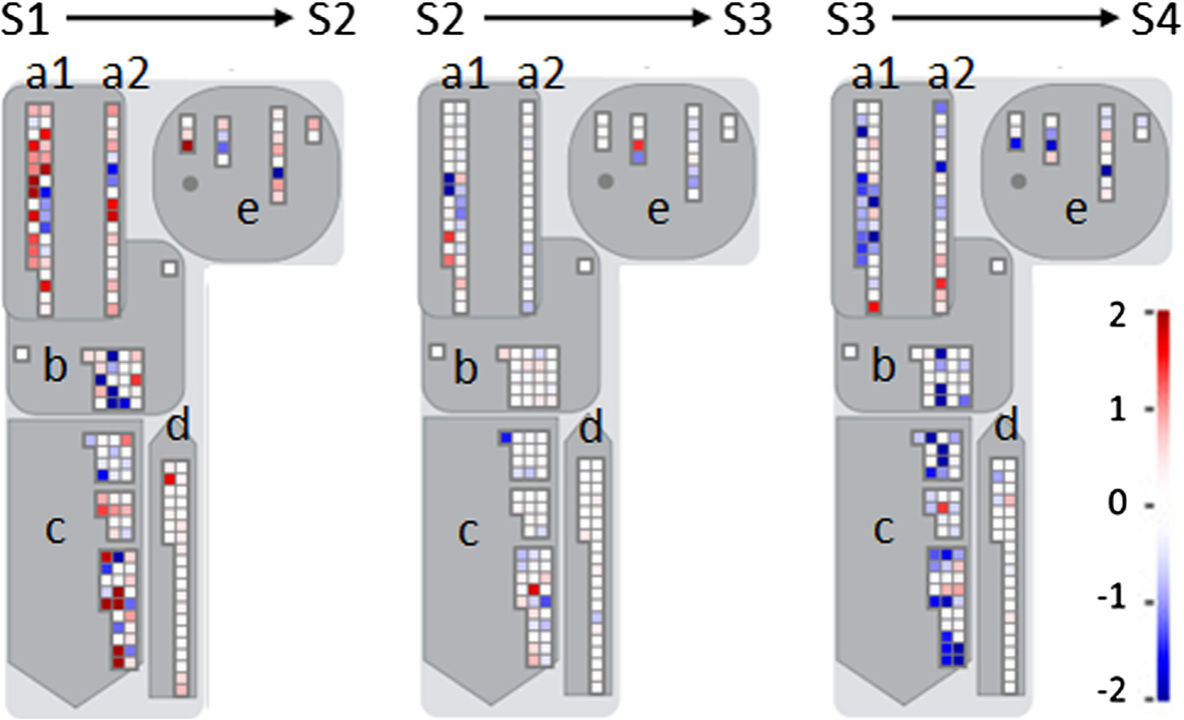
Expression change of unigenes associated with cell wall metabolism in three transitions in Mapman. a1. modification, a2. pectin esterases, b. cellulose syn., c. degradation, d. precursor synthesis, e. cell wall proteins

**Fig. 8:**
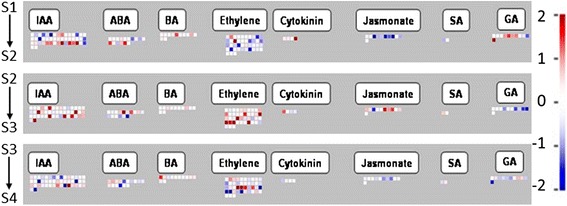
Expression change of unigenes associated with hormone metabolism in three transitions in Mapman. IAA: Auxin/indole-3-acetic acid, ABA: abscisic acid, BA: brassinosteroid*,* SA: salicylic acid, GA: gibberellin

**Fig. 9:**
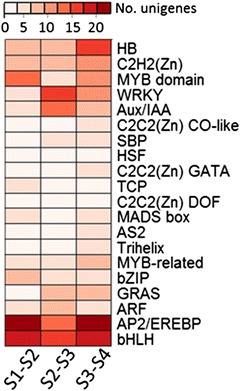
Number of differentially expressed unigenes putatively encoding transcription factors in three transitions

**Fig. 10:**
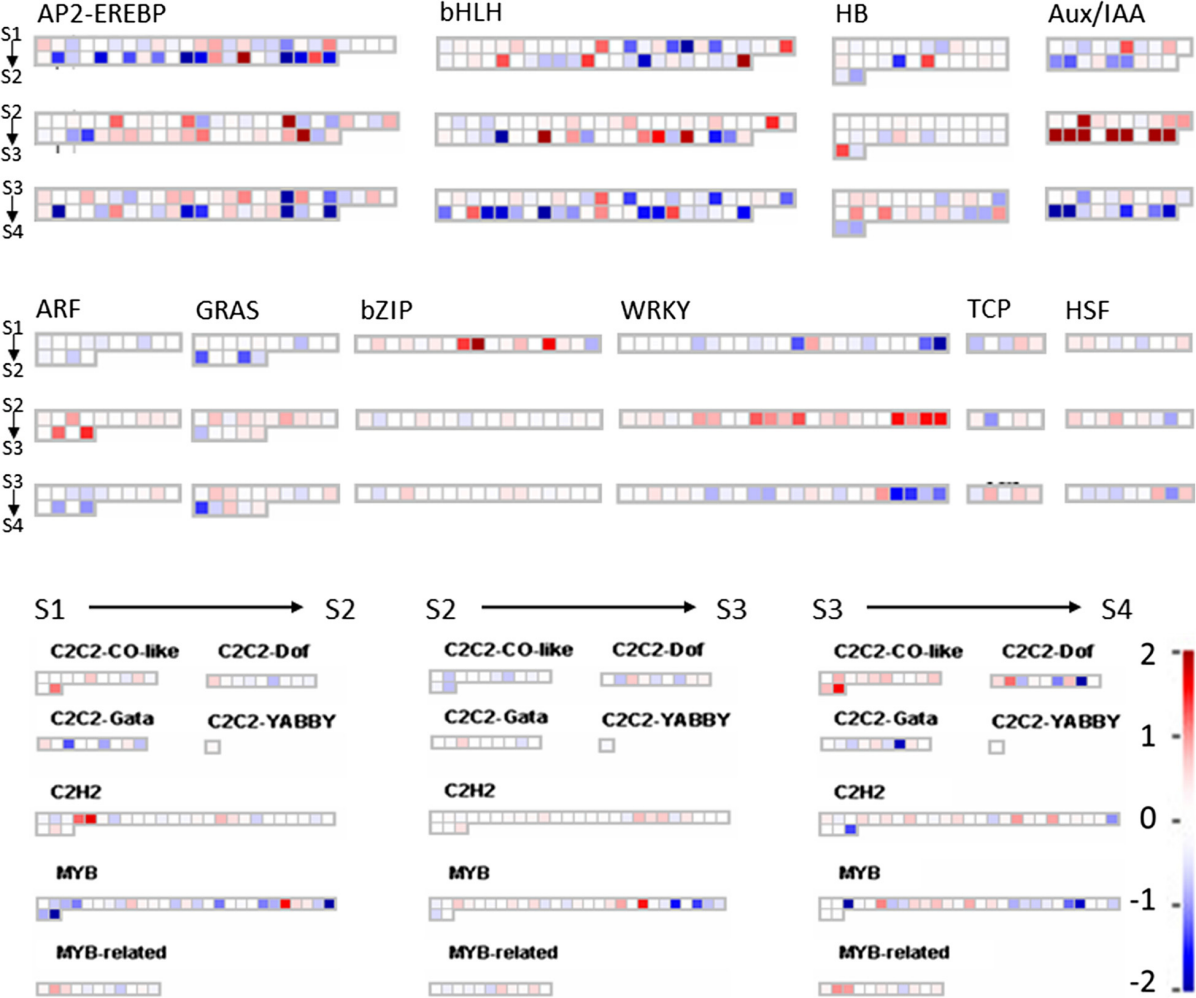
Expression change of unigenes associated with transcriptional regulation in three transitions in Mapman

Given the importance of cell wall metabolism, hormone biosynthesis and signaling, and transcriptional regulation in overall fruit development processes, DE transcripts putatively encoding proteins of these functions in the three transitions S1-S2, S2-S3, and S3-S4 were further investigated with Mapman (Figs. 7, 8, 9, and 10). Herein we mainly discuss transcripts with FC ≥ 1, with FC defined as the base-2 logarithm of the ratio RSEM count in treatment 2/RSEM count in treatment 1 (FC of 1 indicates that the RSEM count in treatment 2 is twice the RSEM count in treatment 1; FC of 0 denotes no change of the RSEM count between two treatments).

#### Cell wall metabolism

The identities of various cell wall metabolism-associated genes that are expressed during fruit development and ripening have been established for a range of species [11]. Our *de novo* pear fruit transcriptome contained 341 transcripts annotated to be associated with cell wall metabolism; of these transcripts, 48.3 % were DE across the four advancing stages of fruit maturity examined in this study. The enrichment of the cell wall category in all three transitions (i.e., S1-S2, S2-S3, and S3-S4) (Additional file 6) supports the idea that cell wall metabolism is critical during pear fruit growth and development.

More DE transcripts putatively encoding different cell wall proteins were identified in the S1-S2 and S3-S4 transitions than in the S2-S3 transition (Additional file 7A). The numbers of the transcripts with FC ≥ 1 were 29 in S1-S2 (74.1 % were up-regulated), 23 in S3-S4 (88.5 % were down-regulated), and 7 in S2-S3 (Table 3). Furthermore, the numbers of DE transcripts encoding proteins of the same cell wall groups such as degradation and modification were larger in the S1-S2 and S3-S4 transitions than in the S2-S3 transition (Table 3, Additional file 7A). These results suggest that transcripts of cell wall-associated genes experienced a more stable period during the S2-S3 transition, as compared to the S1-S2 and S3-S4 transitions, even though the fruit weight and diameter continually increased from S1 to S4 (Table 1).

**Table 3:** Unigenes associated with cell wall exhibiting a fold change ≥1 in at least one transition

GeneID	Accession No.	FCS2/S1	FC_S3/S2_	FC_S4/S3_	Putative description	Mapman subcategory
PcM_60826	GBXL01006883.1		0.06	0.45*		
PcM_53860	GBXL01007382.1	-1.78*	0.51	0.13		
PcM_60371	GBXL01015861.1	-0.36	0.44*	-1.22*	Cellulose synthase	Cellulose synthesis
PcM_60480	GBXL01009925.1	-2.35	0.11	-2.84*		
PcM_61744	GBXL01007569.1	-2.31	-0.15	-3.19*		
PcM_46839	GBXL01044408.1	2.44*	-3.01*	-1.65		
PcM_46838	GBXL01050488.1	2.38*	-2.89*	-1.39		
PcM_49182	GBXL01020463.1	1.94*	-0.27	-0.18		
PcM_38736	GBXL01044154.1	1.16*	-0.18	0.42		
PcM_40371	GBXL01017887.1	1*	-0.51*	0.47*	Xyloglucan endotransglucosylasehydrolase	
PcM_17027	GBXL01027330.1	0.4	-0.14	1.57*		
PcM_32160	GBXL01022618.1	-0.97*	-1.15*	0.75*		
PcM_42818	GBXL01021708.1	-1.05*	-1*	-2.69*		
PcM_44589	GBXL01022862.1	-1.36*	-0.69*	-0.61*		Cell wall modification
PcM_45047	GBXL01021165.1	-1.59*	-0.78	-1.11		
PcM_16347	GBXL01061133.1	1.79*	0.43	-0.91*		
PcM_53964	GBXL01025873.1	1.74*	0.84*	-0.71*		
PcM_53965	GBXL01042551.1	1.69*	0.43	-0.46*		
PcM_54090	GBXL01015731.1	1.61*	-0.01	-0.36*	Expansin	
PcM_12834	GBXL01069216.1	1.35*	1.42*	-1.26*		
PcM_35266	GBXL01087727.1	1.09*	1.21*	-1.28*		
PcM_39832	GBXL01042478.1	0.35	-0.38	-2.56*		
PcM_48614	GBXL01023232.1	0.06	0.45	-3.89*		
PcM_38951	GBXL01069454.1	1.85*	-0.24	-0.82		
PcM_38950	GBXL01048046.1	1.66*	-0.31	-0.88	Pectinesterase	
PcM_51815	GBXL01007030.1	1.04*	-0.38*	-1.19*		Pectin esterase/acetylesterase
PcM_26779	GBXL01016819.1	-1.59*	-0.11	-1.84*		
PcM_59006	GBXL01015518.1	0.88*	-0.45	1.47*	Pectinacetylesterase	
PcM_46568	GBXL01032887.1	1.69*	-0.21	-0.94	Rhamnose biosynthesis	Precursor synthesis
PcM_58600	GBXL01013348.1	1.06*	-0.36	1.4*		
PcM_61178	GBXL01005855.1	1.18*	-0.12	-0.94*		
PcM_55353	GBXL01013717.1	-0.56*	-0.77*	-1.06*	1,4-beta-glucosidase	
PcM_38155	GBXL01035445.1	-1.61*	-0.6	-1.55		
PcM_66369	GBXL01014912.1	0.2	-0.37	-3.31*		
PcM_28397	GBXL01012966.1	-0.8	-0.21	-2.59*	Cellulase	
PcM_58600	GBXL01013348.1	1.06*	-0.36	1.4*	Glycosyl hydrolase	
PcM_66369	GBXL01014912.1	0.2	-0.37	-3.31*		
PcM_41799	GBXL01020764.1	2.71	1.77*	0.88		Degradation
PcM_50534	GBXL01051254.1	-1.43*	-0.74	-1.09		
PcM_43479	GBXL01042875.1	1.91*	-0.83*	-1.32*		
PcM_54883	GBXL01034747.1	2.04*	0.61*	-1.6*	Pectin lyase/Polygalacturonase	
PcM_46178	GBXL01016660.1	-2.44	-0.59	-1.69*		
PcM_54884	GBXL01035215.1	2.18*	0.84*	-1.72*		
PcM_42684	GBXL01015107.1	-1.18	-0.4	-2.04*		
PcM_54851	GBXL01015306.1	0.59	-0.56	-2.18*		
PcM_49140	GBXL01031214.1	2.11*	-0.69*	-1.89*	Pectate lyase	

*the unigene is differentially expressed in the correspondent pairwise analysis (p-value ≤ 0.05)

In the studies of fruit development in several species such as tomato, apple, grapevine, and orange, genes associated with cell wall synthesis, modification, and degradation received a large amount of attention [22, 56–58]. The DE transcripts in these functional categories were considered through the three transitions (Fig. 7, Table 3).

Cellulose synthase contributes to building the cellulose backbone of the plant cell wall [59]. In ‘La France’ pear, the expression of a *cellulose synthase A catalytic subunit* (89 % identical to PcM_60480) was up-regulated from −7 (flower bud) to 30 DAFB, but there were no significant changes in its abundance during later fruit development stages [18]. In our study, the putative *cellulose synthase* gene group was enriched only in the S2-S3 transition (Additional file 6). Moreover, in the S3-S4 transition, three annotated *cellulose synthase* genes (PcM_60371, PcM_60480, and PcM_61744) were down-regulated. Ahmed found that cellulose content did not change during ripening of ‘Bartlett’ pear [60]. Our results therefore suggest that accumulation of putative *cellulose synthase* transcripts and likely more cellulose construction occurred before the S4 stage in ‘Bartlett’ pear. Furthermore, it is interesting to note that a down regulation of putative *cellulose synthase* genes coincided with the fruit's reaching S4 (softened without ethylene treatment). Whether this down-regulation could be a prerequisite for, or a consequence of, attainment of ripening capacity requires further investigation.

Xyloglucan endotransglucosylases/hydrolases (XTHs) are cell wall modification enzymes that are thought to be involved in disassembly of the cellulose-xyloglucan matrix by cleaving the xyloglucan β-D-glucan backbones and then linking xyloglucan segments into them to loosen the cross-links between cellulose [61]. This cell wall-modifying action may contribute to the relaxation of cell wall structures and fruit softening as ripening proceeds. Transcript abundance of most annotated *XTHs*increased from S1 to S2 and thereafter decreased from S2 to S4 (Fig. 7, Table 3). Our expression results for an *XTH* transcript PcM_40371 contrast with earlier findings by Fonseca et al., where a ‘Rocha’ pear homolog (96 % identical to PcM_40371) had low expression during fruit growth (60 to 104 DAFB) and only exhibited an increase in abundance during fruit ripening (3–15 days after harvest at 104 DAFB) [62]. However, consistent with our data, several *XTH* genes were upregulated during tomato fruit growth [63]. Miedes and Lorences also reported an increase of the overall XTH enzyme activity coincident with these *XTH* gene expression changes, suggesting the contribution of XTH to cell wall formation during fruit growth [63]. Therefore, we suggest that *XTH* genes and their enzyme activity play an important structural role in cell wall during the S1-S2 transition.

In addition to XTH, the cell wall modification group contains Exp proteins that have been identified to be involved in polysaccharide association leading to cell wall loosening [64]. The expression of six out of the eight putative *Exp* genes increased from S1 to S2, while the transcript abundance of all eight *Exps* decreased from S3 to S4 (Table 3). These results agree with and complement the report by Hiwasa et al., in which transcripts of *PcExp4* and *PcExp6* (PcM_53964 and PcM_16347 homologs, respectively) were more abundant in young growing fruit than in mature fruit of ‘La France’ pear [65]. This highlighted the important function of some *Exps* in cell wall modification during fruit development. Any loosening of the cell wall caused by Exp proteins also may enhance the abilities of other cell wall-targeting enzymes to move within the apoplast (i.e., diffuse through the porous wall fabric) and, consequently, facilitate fruit softening.

Genes encoding pectin degradation enzymes involved in fruit softening, including pectin lyases/pectate lyases/polygalacturonases (PTs/PGs), have been well characterized in several species, such as tomato, banana, and strawberry [66, 67]. In the present study, this gene group became more enriched in the S1-S2 transition than in the S3-S4 transition (Additional file 6), suggesting that pectin degradation processes become more active once pear fruit approach the mature stage. Consistent with the results found for *PG* transcripts in ‘Rocha’ pear [62], we detected a slight increase in transcript abundance of *Pc-PG1* (PcM_48945) during the S1-S2 transition (FC ≤ 1, data not shown) and *Pc-PG2* (PcM_41799) during the S2-S3 transition (Table 3). The results confirm that the high accumulation of *PG* transcripts does not start until pear fruit near the climacteric onset. However, in contrast to the expression of these *PGs,* several other pectin degradation-related transcripts presented more significant changes: an increase of three out of four DE *PT/PG*-annotated transcripts in the S1-S2 transition and a decrease of all DE *PT/PG*-annotated transcripts in the S3-S4 transition. It was shown that pectin-derived oligomers (PDOs) induced an increase in ethylene biosynthesis in cultured pear fruit cells [68] and that the PDOs that accumulated when tomato fruit started to ripen could stimulate the ripening of tomato pericarp discs cut from mature-green fruit [69]. Therefore, we suspect that pectic oligomers could be produced by the pectolytic enzymes encoded by the genes with high transcript abundance during the S1-S2 and S2-S3 transitions, and these events may contribute to the increased ethylene production at S4 that subsequently lead to the softening of these fruit during 20 °C storage without the need for ethylene treatment. Our results showing high expression of three *PG* genes prior to fruit ripening is the first evidence at the transcript level of possible increases in cell wall degradation enzymes that could generate signal molecules from cell wall fragments to stimulate the development of ripening capacity in European pears.

Additionally, we found similar gene expression patterns of different cell wall functional groups including *Exps* and *PTs/PGs*, which had high levels at S1, S2 and a lower level at S4 (Table 3). The co-expression of these genes may imply the collaboration of these proteins in modifications of complex cell wall polysaccharide networks that are required for fruit cell growth. This finding is similar to what was reported in tomato, in which, compared to the wild type, a significantly greater fruit firmness and reduction in cell wall pectin solubilization and depolymerization was shown in the double suppression line of *LeExp1* and *LePG* but not in the single mutant lines that were tested [70].

#### Hormone biosynthesis and signaling

Hormone-associated genes play important roles in the regulation of ripening capacity [6]. In our *de novo* pear fruit transcriptome, 415 unigenes were annotated as hormone-associated; of these, 35.4 % were DE among the four maturity stages.

In the hormone functional group, the highest number of DE unigenes was associated with auxin (Fig. 8, Additional file 7B). The greatest changes in expression across stage transitions were observed for these unigenes (Table 4), highlighting the potential role of auxin in regulating developmental processes that lead to the attainment of ripening capacity. The transcript abundance of annotated *TIR1* (*Transport Inhibitor Response 1*), which is considered to be a key hormone receptor component in the auxin transduction pathway [71], increased from S1 to S3 and decreased in S4. Additionally, most putative auxin-associated transcripts included *SAUR*s (*Small Auxin Up RNAs*) and *GH3*s, which have been identified as auxin-responsive genes in a wide range of plants [72]. Furthermore, various *GH3* genes were reported to be involved in IAA conjugation in many plant species [73]. Our data showed that the gene expression of several annotated *SAURs* significantly increased during the S1-S2 transition, and putative *auxin-responsive GH3* transcripts were up-regulated in the S2-S3 transition and then down-regulated in the S3-S4 transition (Table 4). Our K-means cluster analysis had also determined that clusters K6 and K8, representing unigenes most highly expressed in S3, were enriched in auxin-associated unigenes (Additional file 5). Auxin is considered a senescence retardant in fruit, and the breakdown of endogenous auxin has been reported to initiate ‘Bartlett’ pear ripening [74, 75]. Moreover, IAA levels declined prior to ripening in tomato, grape, and strawberry fruit [76, 77]. The changes in abundance of auxin-associated transcripts in our data suggest an important function of auxin in the S2-S3 transition in particular, where pear fruit developed a capacity to respond to ethylene and ripen. We postulate that a decrease in auxin levels regulated the pear fruit’s responsiveness to ethylene and that this process occurred prior to autocatalytic ethylene biosynthesis.

**Table 4:** Unigenes associated with hormone metabolism exhibiting a FC ≥1 in at least one transition

GenelD	Accession No.	FC_S2/S1_	FC_S3/S2_	FC_S4/S3_	Putative description	Mapman category
PcM_51866	GBXL01012622.1	-0.18	3.92*	-1.33*	9-cis-epoxycarotenoid dioxygenase	
PcM_29642	GBXL01039210.1	1.61*	-0.52*	0.14	LEA family protein	
PcM_48633	GBXL01029293.1	1.46*	-0.72	0.54	GRAM domain family	ABA
PcM_50680	GBXL01021157.1	-1.24*	1.4*	-0.72	GRAM domain family	
PcM_42482	GBXL01031115.1	0.8*	-1.04*	-1.7*	HVA22	
PcM_47337	GBXL01012930.1	-1.3*	0.92*	-0.66*	IAA-amino acid hydrolase	
PcM_63797	GBXL01017071.1	-2.25*	1.79*	-0.66*	IAA-amino acid hydrolase	
PcM_21098	GBXL01006447.1	1.22*	0.59*	-1.41*	TIR 1	
PcM_13379	GBXL01039261.1	2.51*	-0.51	0.69	SAUR family protein	
PcM_38369	GBXL01033665.1	2.03*	-0.85*	0.54*	SAUR family protein	
PcM_65984	GBXL01027013.1	1.25*	0.34	-0.29	SAUR family protein	
PcM_40146	GBXL01027252.1	1.25*	2.01*	0.17	SAUR family protein	
PcM_38644	GBXL01029510.1	1.05*	-0.41	1.22*	SAUR family protein	
PcM_38326	GBXL01023848.1	-1.32*	0.81	-0.4	SAUR family protein	IAA
PcM_31806	GBXL01043949.1	-1.41*	0.89	0.65	SAUR family protein	
PcM_59684	GBXL01038170.1	1.31	1.73*	0.66*	SAUR family protein	
PcM_47372	GBXL01035990.1	1.68	0.44	2*	SAUR family protein	
PcM_60189	GBXL01006889.1	-1.93*	6.78*	-3.98*	Auxin-responsive GH3 family	
PcM_47706	GBXL01035928.1	-0.92	7.77*	-3.88*	Auxin-responsive GH3 family	
PcM_47707	GBXL01020134.1	-0.24	6.91*	-3.62*	Auxin-responsive GH3 family	
PcM_13948	GBXL01088236.1	0.13	4.9*	-3.4*	Auxin-responsive GH3 family	
PcM_56305	GBXL01014002.1	-1.72*	1.76*	-2.9*	ACS	
PcM_50634	GBXL01013598.1	-0.08	4.55*	-2.68*	ACS	Ethylene
PcM_57563	GBXL01007268.1	-0.68	0.15	-2.07*	Ethylene response sensor	
PcM_16535	GBXL01030409.1	1.51*	0.1	-0.76*	Gibberellin-stimulated transcript 1	
PcM_28946	GBXL01045322.1	1.18*	-1.4*	0.28	Gibberellin-stimulated transcript 1	
PcM_59048	GBXL01059923.1	2.22*	-0.09	-1.79*	Gibberellin-regulated family	GA
PcM_35081	GBXL01021584.1	1.05*	-1.08*	-0.58*	Gibberellin-regulated family	
PcM_40461	GBXL01032298.1	1.02*	0.28*	-1.01*	Gibberellin-regulated family	
PcM_39078	GBXL01047276.1	-0.46	-1.45*	0.66	Gibberellin-regulated family	
PcM_44588	GBXL01011629.1	-2.95*	1.76*	-1.08*	Allene oxide synthase	
PcM_40167	GBXL01025516.1	-1.31*	1.03*	-0.76*	Allene oxide cyclase	
PcM_36557	GBXL01029695.1	-2.03*	1.48*	-1.19*	Allene oxide cyclase	JA
PcM_61989	GBXL01000761.1	-1.39*	0.14*	-0.48*	Lipoxygenase	
PcM_47828	GBXL01004710.1	-2.25*	-1.51*	0.34	Lipoxygenase	

*the unigene is differentially expressed in the correspondent pairwise analysis (p-value ≤ 0.05)

Ethylene is well known to be the main hormone regulating climacteric fruit ripening [6]. Our data showed that while the expression of both DE *ACS* genes increased from S2 to S3 and decreased from S3 to S4 (Table 4), their overall expression was low throughout the four stages considered (RSEM counts ≤ 83, data not shown). Therefore, we suggest that the high FCs of the *ACS* transcripts were probably biased due to their low RSEM counts [78]. The abundance of the *ACO* transcript was slightly decreased in the S1-S2 transition (FC = −0.49), but did not significantly change during the later S2-S3 and S3-S4 transitions. This behavior of the *ACO* gene may explain the physiological data, where a higher internal ethylene concentration and ethylene production rate were detected in the S1 fruit that failed to ripen after 14 days at 20 °C. Fonseca et al. reported ACO activity was below detectable levels in ‘Rocha’ pear during fruit growth [62]. Hence, we conclude that neither the expression changes of *ACO* at the S1-S2 transition nor the ethylene produced at S1 had a significant effect on the ripening capacity of ‘Bartlett’ pear. Moreover, our results on the expression of *ACS* and *ACO* genes agree with an ethylene biosynthesis and action model proposed in tomato, in which autocatalytic ethylene production is initiated by induction of an *ACS* [79, 80].

Ethylene is perceived by protein receptors in plant tissues and this binding inactivates kinase activity of CTR1 (constitutive triple response 1), allowing EIN2 (ethylene insensitive 2) and EIN3 to transduce ethylene signaling [81]. In the present study, transcript abundance of the ethylene receptor *Pc-ERS1a* decreased from S1 to S4 and had a maximum FC of −2.07 in the S3-S4 transition (Table 4). The gene expression of a *Pc-CTR1*, PcM_59353, increased from S1 to S3 (FC_S3/S1_ = 0.93) then stayed at a similar level in S4 (data not shown). Previous studies have shown that a decrease in gene expression of the ethylene receptors *LeETR4* and *LeETR6* increased ethylene sensitivity in tomato [82, 83]. In the present study, S4 fruit were capable of ripening after 14 days without ethylene treatment. Therefore, it appears that the ethylene receptor Pc-ERS1a and ethylene signaling protein Pc-CTR1 are involved in signal transduction of ethylene that consequently activated autocatalytic ethylene production in S4 fruit.

Gibberellin (GA) has been reported to stimulate pericarp growth of pea fruit [84] and silique growth of Arabidopsis [85]. In the present study, GA-associated gene subcategories were enriched at the S1-S2 and S2-S3 transitions (Additional file 6). *GA-stimulated transcripts (GASTs)* are known as targets of GA regulation [86]. Two annotated *GASTs* and three putative *GA-regulated* transcripts were up-regulated from S1 to S2 (Table 4). This may indicate that GAs play a role in fruit growth during the S1-S2 transition.

Jasmonic acid (JA) was suggested to regulate fruit growth in apple [87]. Our data showed that similar to GA-associated transcripts, JA-associated transcripts were enriched at the S1-S2 and S2-S3 transitions (Additional file 6). However, in contrast to the expression patterns of GA-associated genes, transcript abundance of the majority of DE JA-associated transcripts decreased in the S1-S2 transition and increased in the S2-S3 transition (Table 4). These transcripts included three putative *allene oxide synthases* (*AOS*) and an annotated *lipoxygenase*, which encode enzymes involved in jasmonic acid biosynthesis. As AOS is considered to be a rate-limiting step in JA biosynthesis [88], the expression patterns of our JA-associated transcripts complement findings by Kondo et al. in growing apple fruit [87]; there, JA was at a high concentration early in fruit development, decreased, and then increased again. Therefore, JA may be involved in the regulation of pear growth and development through stages S1 to S3.

A key enzyme in ABA biosynthesis, 9-cis-epoxycarotenoid dioxygenase (NCED), has been reported to be associated with ripening of several fruit such as ‘Gold Nijisseiki’ pear and strawberry [89, 90]. In our data, one *NCED* showed a high FC at S2-S3, the transition to ethylene responsiveness (Table 4). However, similar to genes related to ethylene biosynthesis, the expression level was very low in all four stages (S1-S4) (RSEM ≤ 53, data not shown), suggesting a possible bias of high FC. We also found genes associated with ABA such as genes encoding GRAM domain proteins [91] and HVA22 [92]. However, we have not seen clear evidence of the importance of ABA genes in pear growth or in association with the development of ripening capacity. Our results seem to agree with those of an earlier study, where the increase in ABA concentration was merely coincident with ethylene evolution during ripening in ‘Jingbaili’ and ‘Gold Nijisseiki’, Asian pears [89].

#### Transcription factors

Analyzing the expression of genes encoding transcription factors (TFs) help to identify key factors that regulate fruit growth and development, particularly those factors that control the fruit’s response to exogenous ethylene and/or its capacity to soften without ethylene treatment. In the pear fruit, a large number of unigenes in the *de novo* transcriptome (1785) were annotated as TFs. Of these unigenes, 32.0 % were DE across the four maturity stages.

Among all DE transcripts putatively identified as TFs, the AP2/EREBP family members were the most abundant (Fig. 9). Similar to the results obtained from microarray analyses of tomato and peach [56, 93], the gene expression of various putative *EREBPs* was either up- or down-regulated across stage transitions (Fig. 10, Table 5). Annotated *bHLH* transcripts were also highly represented and enriched in all transitions with various expression patterns. Given the broad range of processes affected by AP2/EREBPs- or bHLH-mediated regulation, which includes plant development, primary and secondary metabolism, hormone signaling, and response to biotic and abiotic stresses, as well as the intricate target specificity of each member of these TF families [94, 95], it is not surprising to find such diversity in transcriptional activation or repression across the fruit maturity stages considered in this work.

**Table 5:** Unigenes associated with transcriptional regulation exhibiting a FC ≥1 in at least one transition

GenelD	Accession No.	FC_S2/S1_	FC_S3/S2_	FC_S4/S3_	Transcription factor family
PcM_05337	GBXL01042327.1	-2.51*	0.93	-2.29*	
PcM_13256	GBXL01027276.1	-1.36*	0.83*	-0.33	
PcM_41294	GBXL01026826.1	-1.77*	0.5	-0.83	
PcM_41772	GBXL01034802.1	-1.71*	1.17*	-1.46*	
PcM_41788	GBXL01036010.1	1.04*	0.23	0	
PcM_45640	GBXL01037937.1	2.3*	0.23*	-0.41*	
PcM_46439	GBXL01018984.1	0.6*	-0.42	1.11*	
PcM_46667	GBXL01076709.1	1.35*	-0.83	-0.14	AP2/EREBP
PcM_46760	GBXL01016469.1	0.14	1.24*	0.85	
PcM_49196	GBXL01022640.1	0.27	-0.78*	1.03*	
PcM_49742	GBXL01026828.1	-0.04	0.71	1.08*	
PcM_51776	GBXL01014093.1	-0.5	1.25*	-0.53*	
PcM_53142	GBXL01028427.1	-1.21*	-0.03	-0.68*	
PcM_54496	GBXL01030384.1	-2.65*	0.84	-1.82*	
PcM_56494	GBXL01023972.1	1.12*	-0.94*	-1.24*	
PcM_63505	GBXL01025259.1	-1.46*	0.2	-2.17*	
PcM_18139	GBXL01043693.1	2.28*	0.49*	-1.66*	
PcM_30712	GBXL01016493.1	1.39*	0.32*	-1.27*	
PcM_36078	GBXL01028861.1	-1.85*	1.13	-1.57	
PcM_43050	GBXL01038850.1	-0.48	-1.55*	-0.35	
PcM_44405	GBXL01020945.1	1.33*	-2.17*	-1.81	
PcM_46352	GBXL01020512.1	-0.09	-0.66	-1.82*	
PcM_46628	GBXL01032983.1	-0.45	1.63*	-1.7	
PcM_48210	GBXL01016237.1	0.29	1.48*	0.27	
PcM_49940	GBXL01013876.1	1.25*	-0.22*	1.25*	
PcM_51371	GBXL01034804.1	0.45	-0.78*	1.37*	bHLH
PcM_51372	GBXL01020177.1	0.44	-0.43	1.26*	
PcM_52504	GBXL01025732.1	1.42*	-1*	0.24	
PcM_54799	GBXL01017033.1	-0.26	0.34	-1.2*	
PcM_55022	GBXL01015336.1	-1.27*	0.72	-1.49*	
PcM_55076	GBXL01026920.1	-0.66*	1.01*	-1.04*	
PcM_55271	GBXL01023016.1	-0.78	3.62*	-2.15	
PcM_55855	GBXL01013868.1	-2.07*	0.41	-0.08	
PcM_57931	GBXL01011350.1	-1.28*	0.86*	-1.41*	
PcM_67918	GBXL01013506.1	-1.32*	0.05	-0.78	
PcM_16540	GBXL01054281.1	-0.28	2.44*	-1.18*	
PcM_17596	GBXL01027758.1	-1.29*	3.68*	-1.99*	
PcM_28279	GBXL01047796.1	-1.12	4.29*	-1.49	
PcM_28280	GBXL01056229.1	-0.12	3.52*	-1.85	
PcM_32025	GBXL01036838.1	-0.34	2.42*	-0.67*	Aux/IAA
PcM_38194	GBXL01025223.1	-1.08	9.06*	-5.78*	
PcM_47148	GBXL01024472.1	0.13	1*	-0.45*	
PcM_47475	GBXL01047006.1	-1.07	2.78*	-0.61	
PcM_48272	GBXL01016629.1	1.32*	0.18	0.63*	
PcM_54382	GBXL01013284.1	-0.73*	1.95*	-1.05*	
PcM_38533	GBXL01066258.1	0.06	1.47*	-1.05	ARF
PcM_59593	GBXL01017436.1	-0.07	1.24*	-0.97*	
PcM_33194	GBXL01037175.1	-0.02	-0.04	-1.07*	
PcM_41015	GBXL01049062.1	1.18*	-0.8*	1.64*	
PcM_46524	GBXL01017533.1	0.55	-0.41	1.01*	
PcM_52791	GBXL01005794.1	1.26*	0.03	0.59	
PcM_53620	GBXL01030250.1	-0.27	0.37	-2.17*	Zinc finger
PcM_54994	GBXL01022197.1	-0.01	-0.03	-1.91*	
PcM_55872	GBXL01007344.1	1.72*	0.06	0.29	
PcM_58417	GBXL01002149.1	-0.1	-0.12	1.03*	
PcM_59785	GBXL01047471.1	-0.02	0.52	-1.38*	
PcM_11716	GBXL01035919.1	0.6	-0.1	-1.86*	
PcM_37446	GBXL01061426.1	1.61*	-1.59*	-1.24	
PcM_42937	GBXL01025033.1	-1.18*	0.53	-0.19	
PcM_44410	GBXL01030408.1	-1.15*	-0.21	-0.43	
PcM_46163	GBXL01011717.1	-0.86	0.71	-2.62*	MYB
PcM_46403	GBXL01038893.1	-2.18*	-0.53	-0.7	
PcM_46404	GBXL01065769.1	-2.27*	-0.05	-0.18	
PcM_52004	GBXL01030200.1	-0.06	1.55*	-0.71	
PcM_53948	GBXL01014565.1	-1.15*	0.37	-0.26	
PcM_55469	GBXL01015629.1	0.05	-0.26	1.12*	
PcM_17733	GBXL01083898.1	-2.59*	1.58*	-1.23*	
PcM_19891	GBXL01035302.1	-1.26	1.58*	-0.81	
PcM_45813	GBXL01026944.1	-0.19	0.12	1.01*	
PcM_48928	GBXL01013620.1	-0.32	1.07*	-0.9*	WRKY
PcM_50834	GBXL01013367.1	-0.51	1.28*	-0.81	
PcM_53366	GBXL01019223.1	0.09	1.58*	-1.61*	
PcM_59238	GBXL01041013.1	-1.33*	1.34*	-0.52	
PcM_37832	GBXL01016678.1	-0.3	-0.69	1.15*	
PcM_44708	GBXL01027662.1	-0.46	1.36*	-0.85	
PcM_50305	GBXL01019106.1	1.38*	-0.7*	-0.48	HB
PcM_52074	GBXL01024625.1	0.21	-0.31	1.02*	
PcM_52298	GBXL01015986.1	0.06	-0.38	1.06*	
PcM_52865	GBXL01018633.1	-1.47*	0.71*	-0.34*	
PcM_03380	GBXL01014673.1	3.93*	-0.1	0.08	
PcM_49580	GBXL01020588.1	1.66*	-0.11	-0.29	bZIP
PcM_54944	GBXL01011692.1	1.41*	-0.5*	0.25	
PcM_56708	GBXL01013177.1	0.1	-1.04*	0.87*	TCP
PcM_57292	GBXL01010372.1	-1.31*	-0.8	-1.43	GRAS
PcM_57679	GBXL01005360.1	0.01	1.18*	-0.45	SBP
PcM_35299	GBXL01015905.1	1.15*	0.33	-0.08	MADS

*the unigene is differentially expressed in the correspondent pairwise analysis (p-value ≤ 0.05)

In contrast to the unsystematic behaviors of *AP2/EREBP* or *bHLH* genes, the gene expression of members of *bZIP* (basic region/leucine zipper), *WRKY*, *ARF*, and *Aux/IAA* families showed a more consistent response; i.e., either up or down-regulation, with an enrichment (Fisher’s test, p-value ≤0.05) in a specific transition (Fig. 9, Table 5). In particular, high up-regulation of several putative *bZIP* genes occurred at the S1-S2 transition, compliments the previous results that showed one annotated *bZIP* gene with higher expression in early maturity fruit compared to mature and ripening fruit of *‘*Rocha’ pear (Fonseca et al., 2004). While bZIP TFs have been implicated in the regulation of a wide range of processes including biotic and abiotic stress responses, hormone signaling, and development [96], the concerted expression of a subset of *bZIP* members in this study may point to a common functional feature in fruit development that warrants further investigation. Strikingly, expression of a majority of genes putatively encoding the TF Aux/IAA, ARF, and WRKY were up-regulated in the S2-S3 transition and down-regulated in the S3-S4 transition (Table 5). These concerted *Aux/IAA* and *ARF* gene expression patterns paralleled the transcript abundance changes in auxin-associated genes, which peaked in the S2-S3 transition (Fig. 8, Table 4). These results further underscore the important role of auxin in the development of ripening capacity in response to ethylene; i.e., transition from S2 (fruit treated with ethylene were unable to soften) to S3 (fruit treated with ethylene were able to soften).

## Conclusions

**Fig. 11:**
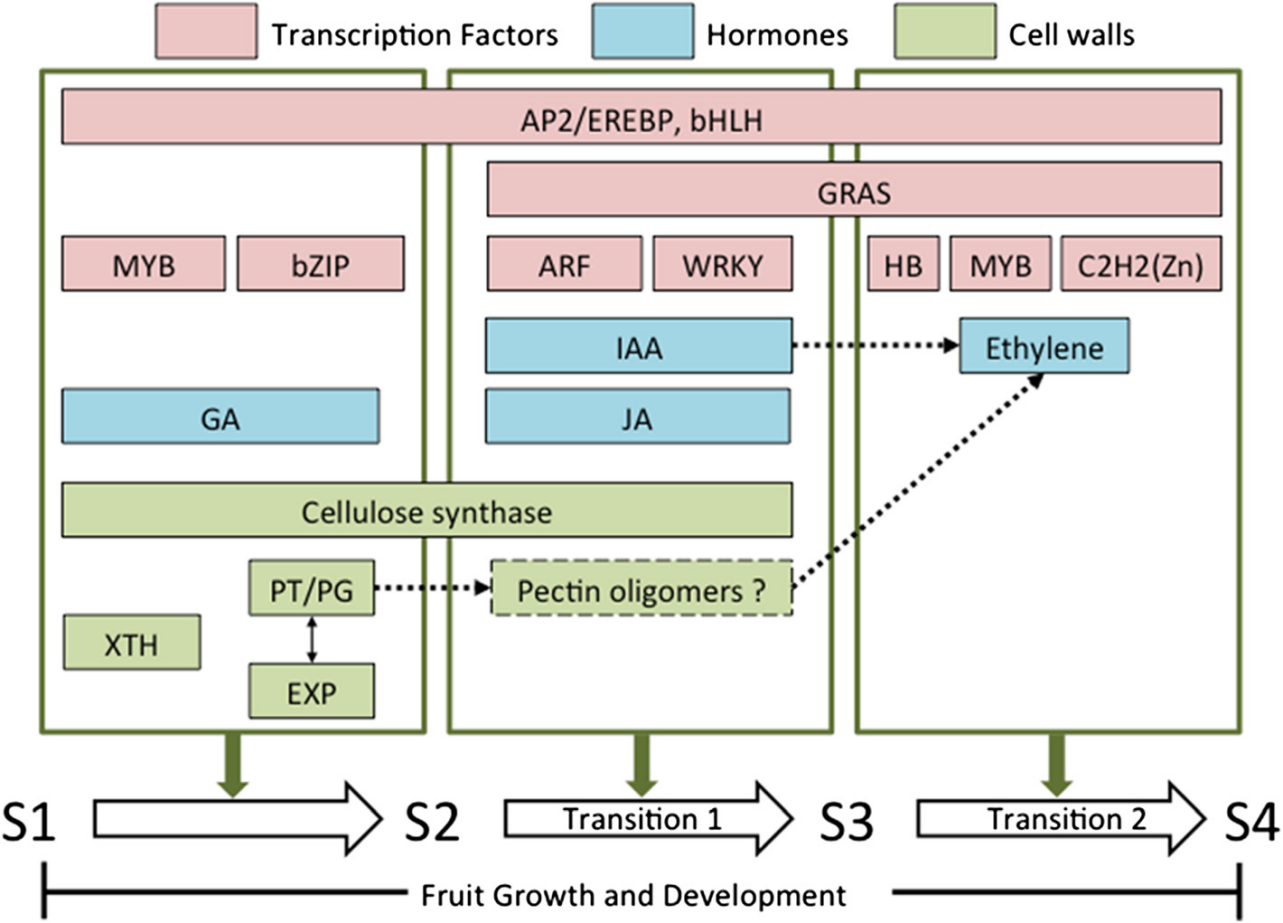
Proposed mechanisms regulating ripening capacity development during the final stages of pear fruit growth. Transition 1: Fruit develop ripening capacity responsive to ethylene treatment; Transition 2: Fruit develop ripening capacity without the need of ethylene treatment. AP2/EREBP: APETALA 2/ethylene response element binding protein, bHLH: basic helix-loop-helix, bZIP: basic region/leucine zipper, ARF: Auxin response factors, HB: homeobox, C2H2(Zn): Cys_2_His_2_ Zinc finger; GA: Gibberellin, JA: Jasmonic acid, IAA: Auxin/indole-3-acetic acid; XTH :Xyloglucan endotransglucosylase/hydrolase , PT/PG: pectin lyase/pectate lyase/polygalacturonase, Exp: Expansin

In this study, we characterized the physico-chemical features and transcriptional profiles associated with the development of ripening capacity in ‘Bartlett’ pear across four maturity stages (S1 through S4). Our analysis, which focused on the differential expression of genes associated with cell wall metabolism, hormone signaling, and transcriptional regulation, suggested a role for specific transcripts, as well as the coordination of members in the same gene family or among gene families, in the attainment of ripening capacity (Fig. 11). We postulate that pectin degradation enzymes may produce early signal molecules (cell wall fragments) that stimulate ethylene biosynthesis associated with the development of fruit ripening capacity. Additionally, auxin-associated genes appear to play an important role in regulating the ability of ethylene-treated fruit to ripen at 20 °C. The transcription factor family bZIP appears to regulate the S1-S2 transition and Aux/IAA, ARF and WRKY may regulate the S2-S3 and S3-S4 transitions. Our results represent a resource for further investigation of some candidate genes or gene groups that regulate the responsiveness of pear, and perhaps other fruit, to ethylene and other plant hormones. In addition, the candidate genes could be examined as molecular markers to indicate the status of ripening capacity, as well as determining appropriate postharvest treatments.

## Additional files

Additional file 1:
**Color changes of pears harvested at four harvest times at harvest and after air/ethylene treatment.** S1, S2, S3, and S4 were harvested a week apart; S4 coincident with commercial harvest. D14: 14 days at 20 °C following treatment of pears with air or 100 μLL^−1^ ethylene (ET) for 24 h. RNA extracted from peel tissues AH of S1 to S4 were used for RNA-sequencing. (The pear image is licensed by http://icons8.com). (PNG 208 kb) Additional file 2:
**Soluble solids content (SSC) and skin color of ‘Bartlett’ pears after 14 days at 20 °C.** Pears were treated with either air or 100μLL^−1^ ethylene (ET) for 24 h at 20 °C. * Mean values with different uppercase letters between Air and ET-treated fruit and different lowercase letters among maturity stages are statistically different according to Tukey’s test (p-value ≤0.05). (PDF 23 kb) Additional file 3:
**Primers for quantitative PCR validation.** (PDF 28 kb) Additional file 4:
**Mapman classification of gene in the** ***de novo*** **transcriptome.** (XLSX 5656 kb) Additional file 5:
**Enriched functional groups in each cluster and enrichment analysis.** * Fisher’s test with Bonferroni correction (p-value ≤0.05). (XLSX 63 kb) Additional file 6:
**Enrichment of groups of Cell Wall, Hormone Metabolism, and Transcription Factors in the three transitions: S1-S2, S2-S3, and S3-S4.** ns: no significant difference, * Fisher’s test with Bonferroni correction. (PDF 29 kb) Additional file 7:
**Number of unigenes associated with A.** cell wall and B. hormones according to Mapman classifications in three transitions: S1-S2, S2-S3, and S3-S4. (PNG 518 kb) Additional file 8:
**Sequences of de no vo transcriptome and their putative functions.** (XLSX 9745 kb)
