# Supplementary figures and images for: A transcriptome approach towards understanding the development of ripening capacity in ‘Bartlett’ pears (Pyrus communis L.)
Source: BMC Genomics. 2015 Oct 9;16:762. doi: 10.1186/s12864-015-1939-9 (PMC4600301; doi:10.1186/s12864-015-1939-9)

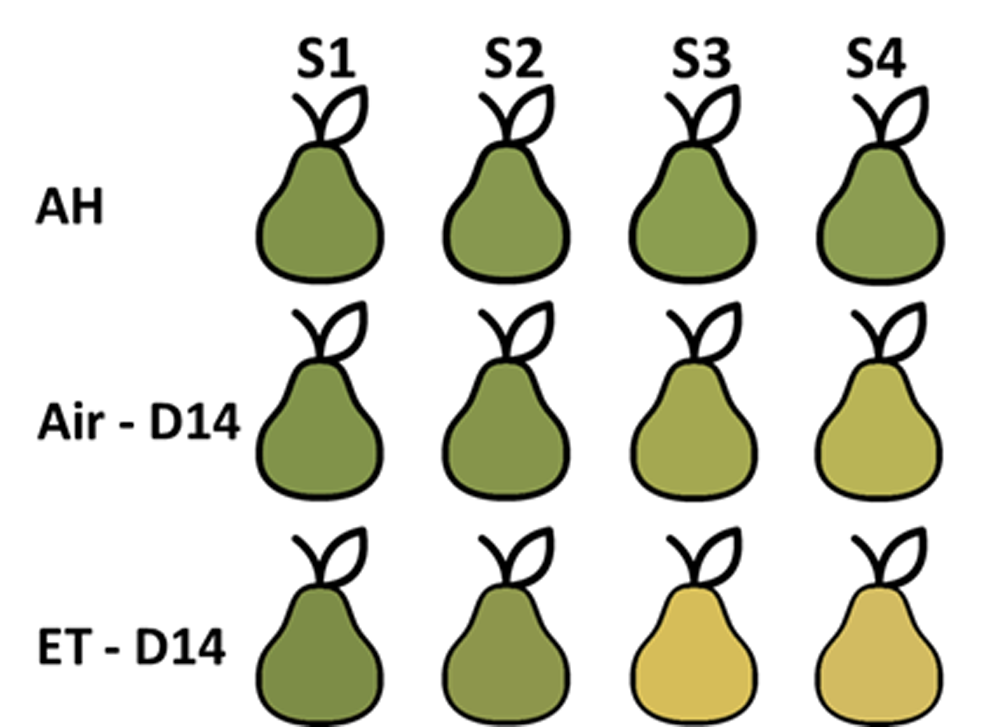

Supplement: Additional file 1: — Color changes of pears harvested at four harvest times at harvest and after air/ethylene treatment. S1, S2, S3, and S4 were harvested a week apart; S4 coincident with commercial harvest. D14: 14 days at 20 °C following treatment of pears with air or 100 μLL−1 ethylene (ET) for 24 h. RNA extracted from peel tissues AH of S1 to S4 were used for RNA-sequencing. (The pear image is licensed by http://icons8.com). (PNG 208 kb) [file 12864_2015_1939_MOESM1_ESM.png]

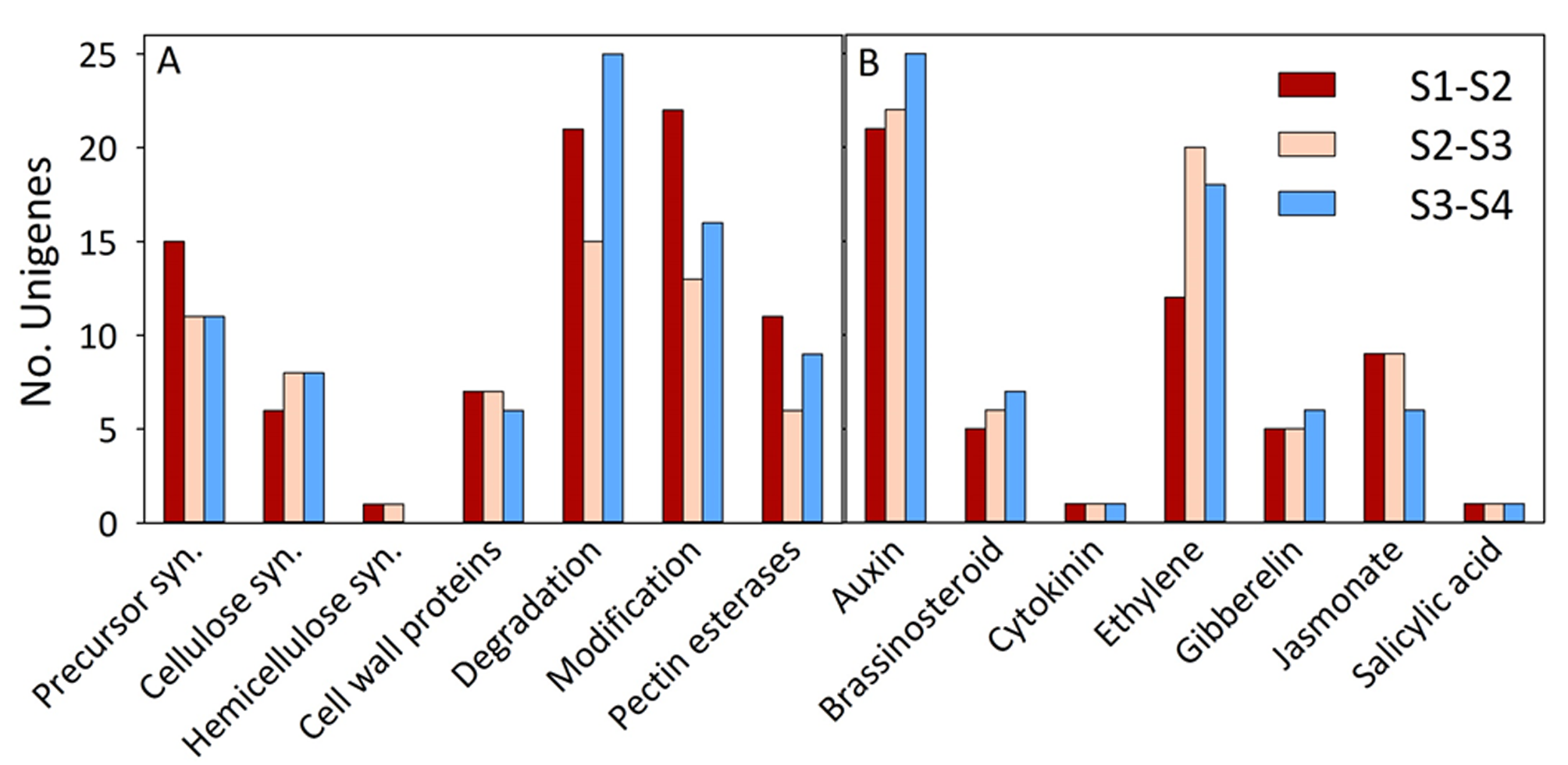

Supplement: Additional file 7: — Number of unigenes associated with A. cell wall and B. hormones according to Mapman classifications in three transitions: S1-S2, S2-S3, and S3-S4. (PNG 518 kb) [file 12864_2015_1939_MOESM7_ESM.png]
