# Supplementary material for: A transcriptome approach towards understanding the development of ripening capacity in ‘Bartlett’ pears (Pyrus communis L.)
Source: BMC Genomics. 2015 Oct 9;16:762. doi: 10.1186/s12864-015-1939-9 (PMC4600301; doi:10.1186/s12864-015-1939-9)
Supplement: Additional file 2: — Soluble solids content (SSC) and skin color of ‘Bartlett’ pears after 14 days at 20 °C. Pears were treated with either air or 100μLL−1 ethylene (ET) for 24 h at 20 °C. * Mean values with different uppercase letters between Air and ET-treated fruit and different lowercase letters among maturity stages are statistically different according to Tukey’s test (p-value ≤0.05). (PDF 23 kb) [file 12864_2015_1939_MOESM2_ESM.pdf]

Maturity Stage	SSC (%)		Skin Color (h°)	
	Air	ET	Air	ET
S1	12.8 Aa	12.3 Aa	116.8 Aa	115.9 Aa
S2	11.5 Ab	11.9 Aa	115.2 Aa	112.5 Aa
S3	12.3 Aab	11.8 Aa	108.7 Ab	93.9 Bb
S4	12.7 Aa	12.4 Aa	103.8 Ac	93.6 Bb
