# Supplementary material for: A transcriptome approach towards understanding the development of ripening capacity in ‘Bartlett’ pears (Pyrus communis L.)
Source: BMC Genomics. 2015 Oct 9;16:762. doi: 10.1186/s12864-015-1939-9 (PMC4600301; doi:10.1186/s12864-015-1939-9)
Supplement: Additional file 3: — Primers for quantitative PCR validation. (PDF 28 kb) [file 12864_2015_1939_MOESM3_ESM.pdf]

Gene ID	Forward 5'- 3'	Reverse 5'- 3'	Putative Function
PcM_40146	GCGGTTTGACGTTGATGTCC	ATCCCCTGCAGTGAAGACAC	SAUR-like auxin-responsive
PcM_43735	ACATGAAGGGTGACGAGGAG	ACCGTTATCTCGCTGACCAC	F-box
PcM_46352	CTCTCTGCCATGGAAACACC	TGGCATCAATGATCTGAGGA	bHLH
PcM_51372	TGCCAGAGTCAATCCTTGCC	ACTCTTCACAGAGAGGCAGTC	bHLH
PcM_57178	ACCTCGGTCTGCTTCAAGTG	TGATGGGAGGAGGAAGATTG	Pectin/Pectate lyase
PcM_57988	CGTTGAAATCCGAATGAGGT	TACTGGGTTTGCGAGTCCTT	Auxin-responsive factor
PcM_60189	CTTTTCCGCACTGTTTGGAC	TACGGTTCCTCCAGCTCAAC	Auxin-responsive GH3
PcM_60321	TGGATGTGTATTGCCCTCCT	TAGAGACGGATGCACACGAG	EIN3-binding F-box
PcM_61061	ACCCTCCACTTGCTGTCATC	TCCATTACGCTCTTGTGTCG	SAM methyltransferases
PcM_61200	ACCAGGCTTGACGATACCAG	AGGTCCACCAACCTTGACTG	Internal control - EF1alpha, AY338250 (GeneBank)
PcM_63124	CCGATCTCTTCCAGTTCCAC	GATGTCACACAGATGCCTACG	Flowering promoting factor 1
PcM_67803	CCTCCACATTGCTTGTTTCC	GATCGTGCATGTTCACCTTG	TIR-NBS-LRR class
