# Supplementary material for: A transcriptome approach towards understanding the development of ripening capacity in ‘Bartlett’ pears (Pyrus communis L.)
Source: BMC Genomics. 2015 Oct 9;16:762. doi: 10.1186/s12864-015-1939-9 (PMC4600301; doi:10.1186/s12864-015-1939-9)
Supplement: Additional file 6: — Enrichment of groups of Cell Wall, Hormone Metabolism, and Transcription Factors in the three transitions: S1-S2, S2-S3, and S3-S4. ns: no significant difference, * Fisher’s test with Bonferroni correction. (PDF 29 kb) [file 12864_2015_1939_MOESM6_ESM.pdf]

Mapman Category	P-value*		
	S1-S2	S2-S3	S3-S4
cell wall	9.914E-40	1.118E-25	2.77E-29
cell wall.precursor synthesis	1.81E-06	1.33E-03	2.63E-02
cell wall.cellulose synthesis	ns	1.42E-02	ns
cell wall.degradation	1.45E-07	3.29E-04	5.93E-10
cell wall.degradation.pectate lyases and polygalacturonases	3.80E-04	3.75E-05	2.82E-06
cell wall.modification	1.76E-16	6.09E-07	2.98E-08
cell wall.pectin*esterases	2.57E-04	ns	4.52E-02
hormone metabolism	5.99E-18	2.15E-32	9.28E-21
hormone.abscisic acid	1.61E-02	2.16E-05	ns
hormone.abscisic acid.induced-regulated-responsive-activated	1.07E-05	1.60E-03	ns
hormone.auxin	2.12E-05	8.48E-08	2.78E-07
hormone.auxin.synthesis-degradation	4.69E-03	ns	9.06E-03
hormone.auxin.induced-regulated-responsive-activated	1.44E-02	3.69E-05	6.48E-04
hormone.ethylene	ns	9.52E-08	6.24E-04
hormone.ethylene.synthesis-degradation	ns	1.37E-07	7.02E-04
hormone.gibberelin.induced-regulated-responsive-activated	2.69E-02	8.95E-03	ns
hormone.jasmonate	1.14E-02	1.95E-03	ns
hormone.jasmonate.synthesis-degradation	9.22E-03	1.42E-02	ns
RNA	2.76E-45	6.75E-34	1.99E-61
RNA.regulation of transcription.GRAS	ns	ns	1.03E-02
RNA.regulation of transcription.HB	ns	ns	1.72E-05
RNA.regulation of transcription.MYB domain	1.44E-02	ns	ns
RNA.regulation of transcription.AP2/EREBP	1.99E-09	2.65E-03	2.60E-09
RNA.regulation of transcription.WRKY domain	ns	5.34E-09	ns
RNA.regulation of transcription.bZIP	3.50E-02	ns	ns
RNA.regulation of transcription.ARF	ns	3.27E-05	ns
RNA.regulation of transcription.Aux/IAA	ns	1.27E-11	ns
RNA.regulation of transcription.bHLH	3.01E-06	4.84E-06	2.03E-05
